# Direct nanoscopic observation of plasma waves in the channel of a graphene field-effect transistor

**DOI:** 10.1038/s41377-020-0321-0

**Published:** 2020-06-04

**Authors:** Amin Soltani, Frederik Kuschewski, Marlene Bonmann, Andrey Generalov, Andrei Vorobiev, Florian Ludwig, Matthias M. Wiecha, Dovilė Čibiraitė, Frederik Walla, Stephan Winnerl, Susanne C. Kehr, Lukas M. Eng, Jan Stake, Hartmut G. Roskos

**Affiliations:** 1 Physikalisches Institut, Johann Wolfgang Goethe-Universität, Max-von-Laue-Str. 1, D-60438 Frankfurt am Main, Germany; 2 Institut für Angewandte Physik, Technische Universität Dresden, Nöthnitzer Str. 61, D-01187 Dresden, Germany; 3 Department of Microtechnology and Nanoscience, Chalmers University of Technology, SE-41296 Gothenburg, Sweden; 4 Institute of Ion Beam Physics and Materials Research, Helmholtz-Zentrum Dresden-Rossendorf, Bautzner Landstraße 400, D-01328 Dresden, Germany; 5 Complexity and Topology in Quantum Matter (CT.QMAT), Cluster of Excellence EXC 2147, Dresden/Würzburg, Germany; 6 Present Address: Department of Electronics and Nanoengineering, Aalto University, Tietotie 3, 02150, Espoo, Finland

**Keywords:** Optical properties and devices, Terahertz optics

## Abstract

Plasma waves play an important role in many solid-state phenomena and devices. They also become significant in electronic device structures as the operation frequencies of these devices increase. A prominent example is field-effect transistors (FETs), that witness increased attention for application as rectifying detectors and mixers of electromagnetic waves at gigahertz and terahertz frequencies, where they exhibit very good sensitivity even high above the cut-off frequency defined by the carrier transit time. Transport theory predicts that the coupling of radiation at THz frequencies into the channel of an antenna-coupled FET leads to the development of a gated plasma wave, collectively involving the charge carriers of both the two-dimensional electron gas and the gate electrode. In this paper, we present the first direct visualization of these waves. Employing graphene FETs containing a buried gate electrode, we utilize near-field THz nanoscopy at room temperature to directly probe the envelope function of the electric field amplitude on the exposed graphene sheet and the neighboring antenna regions. Mapping of the field distribution documents that wave injection is unidirectional from the source side since the oscillating electrical potentials on the gate and drain are equalized by capacitive shunting. The plasma waves, excited at 2 THz, are overdamped, and their decay time lies in the range of 25–70 fs. Despite this short decay time, the decay length is rather long, i.e., 0.3-0.5 μm, because of the rather large propagation speed of the plasma waves, which is found to lie in the range of 3.5–7 × 10^6^ m/s, in good agreement with theory. The propagation speed depends only weakly on the gate voltage swing and is consistent with the theoretically predicted $\frac{1}{4}$ power law.

Plasma waves in solid-state materials and device structures have regained much attention with the emergence of graphene and the direct observation of plasmon polariton excitation and propagation on this two-dimensional material at infrared frequencies^1,2^. The capability to control the carrier density and thus the plasma frequency by doping as well as by the application of a gate voltage allowed the discovery of a plethora of physical phenomena related to plasmons, such as plasmon–phonon coupling^3^, strong electronic correlations measured via the precise determination of the propagation velocity of the plasmons^4^, negative local resistance^5^, and material interactions that limit the propagation distance of the plasmon polaritons^6^. Plasma-wave excitations are also expected to play a significant role in graphene field-effect transistors (FETs), which are widely explored for the detection of terahertz radiation^7–9^. This formation of plasma waves is not specific to graphene but is expected for any type of gated two-dimensional electron gas (2DEG) in the channel of an FET. Such channel plasma waves have gained considerable attention, as FETs became of practical interest for detection^10–13^ and generation^14–17^ of electromagnetic waves at THz frequencies. In contrast to the plasmons of 2DEGs of pure materials, one expects here the development of gated plasma waves^4^, which collectively involve the charge carriers of both the 2DEG and the gate electrode and exhibit a typical polariton-like dispersion relation.

In the following, we explore the case of THz detection. At lower frequencies, whenever an electromagnetic wave is injected into the channel of an FET (either from the source-gate port or from the drain-gate port), the 2DEG responds in a quasi-static manner, i.e., without any phase delay along the channel. This holds true as long as the wavelength *λ*_*pl*_ of the plasma wave at that frequency is much larger than the channel length *L*. With $\omega = v_{pl}\cdot 2\pi /\lambda _{pl}$, where *ν*_*pl*_ denotes the gate voltage-dependent propagation velocity of the plasma wave^14,18^, this leads to the condition $\omega \ll 2\pi \,v_{pl}/L$. Whenever this condition is not fulfilled, for instance at a higher angular frequency *ω* of the electromagnetic wave, the 2DEG responds in a wave-like manner. Theory predicts that a plasma wave is launched at the coupling port and propagates along the channel. The transition from the quasi-static to the plasma-wave regime occurs over a larger frequency range, typically around frequencies of hundreds of GHz to several THz for the case of typical antenna-coupled FET THz detectors (TeraFETs)^13,19–21^.

The strongest experimental evidence for the excitation of plasma waves in these detectors is the observation of a so-called plasma-resonant behavior in devices with high charge-carrier mobility at cryogenic device temperatures^22–26^. One finds a modulation of the rectified voltage or current upon the changes in gate voltage or radiation frequency. This can be understood to stem from the formation of standing plasma waves provided they live long enough to be reflected at the end of the channel. At room temperature, there is new evidence coming from graphene amplifier devices^27^. For detectors, the evidence for plasma wave excitation is more indirect and hence weaker, and comes mainly from the following aspects.

At first, the impedance of the channel has been predicted to change significantly with frequency upon reaching the plasma-wave regime^21,28^. This prediction has led some THz detector designers to account for plasma waves via waveguide transport models^28–30^. To what extent the very good performance of the new detectors^19,21^ can be attributed to this design aspect is, however, not sufficiently clear and experimentally still open to be shown.

Second, sensors for either the intensity gradient or the polarization helicity of THz beams have been developed on the hypothesis of the existence of plasma waves in the transistor channels^31,32^, but their operation can also be explained, at least qualitatively, by conventional resistive mixing, including capacitance effects.

What has been missing until now is a direct visualization of plasma waves in a TeraFET channel during the THz detection process. In this letter, we report on this direct observation at room temperature using antenna-coupled graphene field-effect transistors (GFETs) exposed to THz radiation from a free-electron laser source. Similar to ref. ^4^, we exploit the fact that GFETs can be fabricated with a buried gate electrode/gate oxide stack and a graphene sheet on top, hence leaving the 2DEG in graphene accessible for measurements. This design allows us to observe the plasma waves in the channel with the help of a scattering-type scanning near-field optical microscope (s-SNOM) operated at THz frequencies. Although the plasma waves in this open system are strongly damped, we are able to determine their decay length and propagation speed as a function of gate voltage. The obtained values are in excellent agreement with the values in literature, which confirms the direct observation of plasma waves in the FET channel.

Figure 1a shows a photograph of our GFET device monolithically integrated with a bow-tie antenna. The design follows that of the graphene TeraFET detector presented in ref. ^9^, except for the Ti/Au gate electrode (thickness: 22 nm) that now lies under a sheet of monolayer graphene, separated from this sheet by a 25-nm-thick *Al*_2_*O*_3_ gate dielectric. The fabrication details can be found in the Supplementary Materials. Figure 1b displays a scanning electron micrograph, and Fig. 1c shows a schematic representation of the channel region of the TeraFET device. The buried gate metallization is connected to one arm of the bow-tie antenna. This arm is split into two parts and embeds the drain electrode. The ensuing capacitive coupling of the gate and drain sets them on the same high-frequency electrical potential. Because of this shunting effect, THz waves are expected to be injected only from the source side into the FET channel, thus ensuring the asymmetric wave coupling required for efficient rectification^28,33^.

**Fig. 1:**
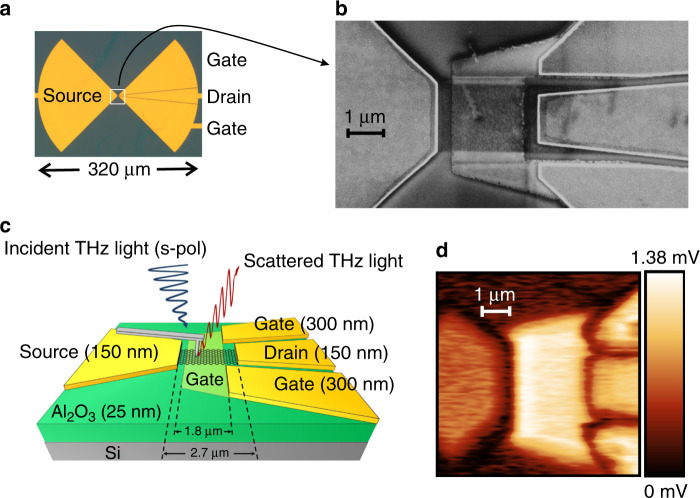
Graphene TeraFET detector. **a** Microscope image of the device showing the bow-tie antenna. **b** Scanning electron micrograph of the apex region of the antenna arms (region marked by the white box in **a**) showing the FET channel. The graphene layer on top of the buried gate can be clearly identified. **c** Schematic view of the channel region between the drain and source electrodes, specifying the thicknesses of the gold and Al_2_O_3_ layers. Also indicated is the cantilever of the s-SNOM. The cantilever arm is almost parallel to the symmetry axis of the antenna leaves. **d** Single-scan s-SNOM near-field image of the field distribution upon illumination with radiation at 2 THz (total time-averaged power: 45 mW, beam diameter: 1 mm), 2-Ω-demodulation, gate voltage *V*_*g*_ = 0 V, drain-source voltage *V*_*ds*_ = 0 V and lock-in integration time of 50 ms. The color scale represents the output voltage of the lock-in amplifier, which is proportional to the local field amplitude

Figure 1c also contains a schematic representation of the s-SNOM cantilever mimic with the metallized probe needle. The THz radiation impinged at an angle of 55° relative to the surface normal with its phase fronts and polarization oriented parallel to the antenna’s symmetry axis (s-polarization). The THz radiation scattered by the probe tip passed through a polarization filter, set to let only p-polarized scattered light pass into the detector (cross-polarization detection technique^34^ for an improved suppression of the background radiation). The s-SNOM was operated in power-detection mode (self-homodyne detection mode^35^), and the near-field signal was extracted by higher-harmonic demodulation^36,37^.

Figure 1d displays a near-field image of the gate region for the device illuminated by radiation at 2 THz (for the selection of that frequency, see the Supplementary Materials). The detector signal was demodulated at the second harmonic of the cantilever oscillation frequency Ω (for the topographic image recorded simultaneously by the atomic force functionality of the setup, see Fig. S10 in the Supplementary Materials). The brightness of the near-field signal in Fig. 1d is dependent on the amplitude of the local electric field (see Eq. (7) in the Supplementary Materials). The signals on the gate, drain, and source electrodes represent the amplitude of the time-averaged THz waves arriving from the antenna arms. The signal strengths on the drain and gate electrodes are approximately equal, which is consistent with their capacitive coupling. The source contact exhibits a weaker signal, which we attribute to a non-symmetric illumination of the bow-tie antenna. The highest field amplitude is observed on the channel. It peaks at the source side and gradually reduces to a pedestal towards the drain side. As we discuss further in the following, this gradient is consistent with the injection of an overdamped plasma wave from the source onto the channel. This wave signal superimposes the THz signal of the gate metal under the graphene, which is only partially screened by the gate oxide and the graphene sheet^38–40^. We note that plasma wave injection from the source side is expected because of the capacitive shunting of the gate and drain, as stated above. We find no indication for wave injection from the drain side, although the field amplitude on that side of the antenna is higher than on the source side.

For a careful analysis of the plasma wave signature in the channel, we recorded line scans along the channel instead of full images and measured the traces with a longer lock-in integration time constant (1000 ms instead of 50 ms, as for the images) and as a function of the gate voltage *V*_*g*_. All traces shown in the following represent the averages of three scans, in the forward direction, with the individual scans exhibiting high reproducibility. No drain-source bias voltage was applied (corresponding to the usual bias-free operation of TeraFET detectors, which ensures their low-noise performance^13,19–21^). As the graphene layer was exposed to air as well as to the THz radiation field, we examined the functionality and integrity of the THz-illuminated TeraFET continuously by measuring the DC drain-source current *I*_*ds*_ as well as the rectified current *I*_*rect*_ every few minutes. Figure 2a displays two exemplary $I_{ds}\left( {V_g} \right)$ curves of one of the devices (termed “Device 1”). The charge neutrality point (CNP, Dirac point), identified by the minima in the curves, was at $V_{g,CNP} = 3.2\,{\mathrm{V}}$. We note that the value of *I*_*ds*_ at the CNP is rather large, which is indicative of pronounced potential fluctuations over the area of the gate. A pair of $I_{rect}\,\left( {V_g} \right)$ curves are displayed in the inset of Fig. 3a and are discussed below.

**Fig. 2:**
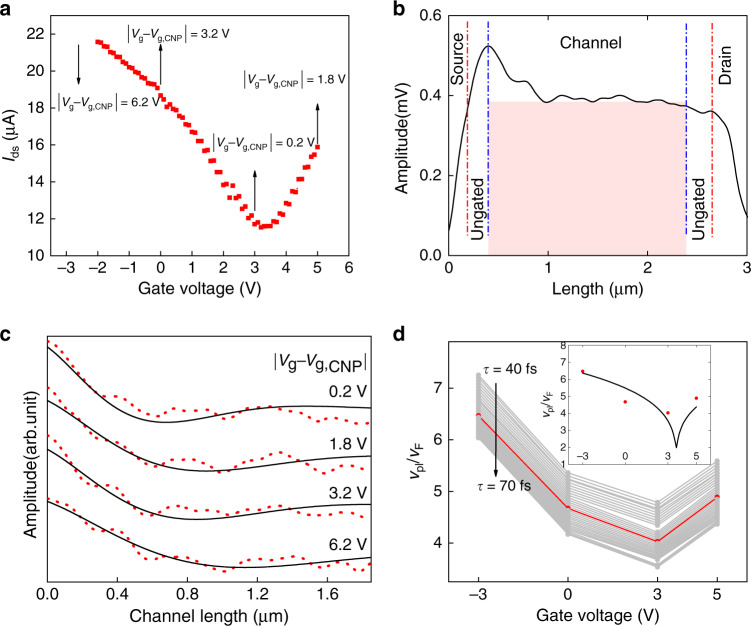
Device 1. **a** Drain-source current of the TeraFET $\left( {V_{ds} = - 0.1\,{\mathrm{V}}} \right)$ when illuminated with 45mW of radiation at 2 THz (two traces). The arrows mark the voltage positions where s-SNOM line scans were performed. **b** 3Ω-near-field trace along the channel between the source and drain. Red dash-dotted vertical lines demark the edges of the source and drain electrodes; blue dash-dotted lines, the beginning and end of the gate electrode. The signal contribution attributed to the amplitude of the THz potential of the gate metallization is indicated by the colored rectangle. **c** Red-dotted lines: Near-field traces (zoomed-in on the plasma-wave component of the signal) for selected gate voltages indicated by the arrows in **a**. The curves are arranged in sequence of increasing value of $|V_g - V_{g,CNP}|$ from 0.2 V to 6.2 V. The curves are shifted vertically for clarity. Full black lines: calculated traces based on the fit function and the boundary conditions given in the Supplementary Materials (fit function: Eq. (7)). **d** Extracted *ν*_*pl*_ for values of *τ* between 40 fs and 70 fs. The red line represents *ν*_*pl*_ for *τ* = 55 fs. The inset compares the extracted values of *ν*_*pl*_ for *τ* = 55 fs (red dots) with the predictions of the theoretical model of refs. ^18,43^

**Fig. 3:**
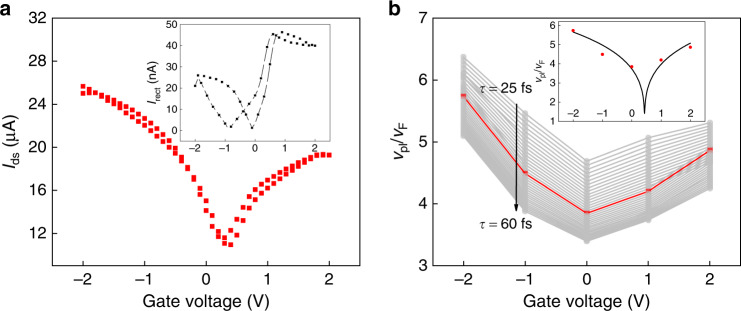
Device 2. **a** DC drain-source current as a function of the gate voltage; the measurements were made with the device illuminated at 2 THz $\left( {V_{ds} = - 0.1\,{\mathrm{V}}} \right)$. Inset: Absolute value of the rectified current as a function of the gate voltage (no drain-source bias voltage). The direction of the current flow changes at zero values of the current. **b** Extracted *ν*_*pl*_ values for *τ* between 25 fs and 60 fs. Red dots depict *ν*_*pl*_ for *τ* = 40 fs. The inset compares the extracted values of *ν*_*pl*_ fo *τ* = 40 fs with the theoretical predictions (cf. Figure 2d)

Figure 2b displays an s-SNOM line trace recorded at the third harmonic of the cantilever oscillation frequency for $V_g = 0\,{\mathrm{V}}$. The signal peaks at the source-side edge of the channel and decreases to a pedestal towards the drain. The source-side maximum reproduces the peak feature already seen in the image of Fig. 1d. As stated above, it is plausible to assume that the total measured signal is the sum of a large contribution by the THz signal on the gate electrode—only partially screened by the graphene sheet—and a smaller signal by the plasma wave injected from the source side onto the gated graphene. The former contributes a spatially constant pedestal on which sits the decaying signal from the plasma wave. The additional weaker modulation seen on top may result from spurious standing-wave artefacts involving background reflections, which can occur anywhere in the beam path^41,42^. Another very plausible explanation is scattering of the plasma wave by surface contamination; this option is explored more in the Supplementary Materials.

The red-dotted curves of Fig. 2c represent line scans of the gated region for four values of *V*_g_: −3, 0, 3, and 5 V. The data for $V_g = 0\,{\mathrm{V}}$ are those already displayed in Fig. 2b but now with the focus zoomed-in on the plasma-wave contribution. The curves for the other gate voltages, shown in sequence of increasing value of $|V_g - V_{g,CNP}|$, are vertically displaced for clarity. Although the traces look rather similar, they exhibit a slight shape variation, most noticeable in the slope of the decaying signal immediately after the maximum at the source side of the channel. This variation is indeed expected because of the dependence of the propagation velocity *ν*_*pl*_ on the average sheet density $\bar n$ of the charge carriers^18,43^. The functional dependence is fairly weak, with $v_{pl} \propto \bar n^ {\frac{1}{4}}$. $\bar n$ itself depends on the gate voltage. For ideal gated graphene, one finds $\bar n \propto \left| {V_g - V_{g,CNP}} \right|,$ but potential fluctuations (caused, for example, by adsorbed molecules and variations in the properties of the interface between the graphene sheet and the gate insulator) weaken this dependence, notably in the vicinity of the CNP.

We simulated the traces by an s-SNOM detection model assuming that a plane plasma wave is launched at the source-sided edge of the gate, propagates towards the probe tip, and then interferes with the incident field at the tip position (for details, see the Supplementary Materials). s-SNOM detects a time-averaged scattered signal proportional to the power of the interfering waves at a higher harmonic of Ω. The unknown model parameters are the propagation velocity *ν*_*pl*_ and the damping time *τ* of the plasma wave. In accordance with the literature on air-exposed graphene, the latter is assumed to lie in the range of 25–70 fs^44–46^. For any value of *τ* chosen in this range, we fitted the s-SNOM response function derived in the Supplementary Materials (Eq. (7) together with Eq. (4)) to the measured data, obtaining a best value of *ν*_*pl*_ (with a narrow error range, indicating the reliable fitting; see Supplementary Materials). Figure 2c displays the respective fitted curves for $\tau = 55\,{\mathrm{fs}}$. With $\omega \tau = 0.69$, we are still in the overdamped wave regime (characterized by $\omega \tau \,<\, 1$), and a second lobe of the wave is only barely observable. Remarkably, the overdamped waves still travel a considerable distance because of their high propagation velocity. The values of *ν*_*pl*_ are displayed in Fig. 2d as functions of the values of *τ* in the range from 40 fs to 70 fs and relative to the Fermi velocity *v*_*F*_ = 1 µm/ps. *ν*_*pl*_ has a minimum at $V_g = 3\,{\mathrm{V}}$, the gate voltage of the measurements closest to $V_{g,CNP}$. Away from this voltage, the propagation speed is larger, as expected from theory. The inset of Fig. 2d compares the extracted values of *ν*_*pl*_ for $\tau = 55\,{\mathrm{fs}}$ with the theoretical values derived from Eq. (17) of ref. ^18^ for ideal gated graphene (assuming a thickness of the gate dielectric of 25 nm). The agreement is good except in the vicinity of the CNP, where the average carrier density of a real device does not decrease as much as theory predicts for the ideal case. This outcome is expected because of the local fluctuations in the graphene’s electrical potential mentioned in the discussion of Fig. 2a, influenced by local variations in the interface properties, surface contaminations, and adsorbed air molecules.

Figure 3 displays corresponding data for a second device of nominally identical structure but fabricated in an independent process run (“Device 2”). The $I_{ds}\,\left( {V_g} \right)$ curve in Fig. 3a shows that the CNP voltage is different from that of Device 1, which emphasizes the sensitivity of graphene’s electrical potential to the fabrication details and the air-exposure history. The inset of Fig. 3a displays an exemplary curve of $I_{rect}\left( {V_g} \right)$, the rectified current. Its functional dependence is as expected for rectification by the Dyakonov–Shur mechanism in combination with the hot-carrier thermoelectric effect^8,9^. The hysteretic behavior for up- and down-sweeps of *V*_*g*_ is evidence of a significant influence of charge-carrier trapping effects on the electric potential of the device. The optical current responsivity (rectified current vs. impinging optical power, not taking the coupling efficiency of the radiation into account) reaches a maximum of $2\frac{{\mu A}}{W}$, much lower than the value of $0.5\frac {{mA}}{W}$ of a top-gated graphene TeraFET of similar layout but optimized for its responsivity and equipped with a substrate lens for in-coupling of the THz radiation^9^. Figure 3b displays the results of the evaluation of the s-SNOM line-scan measurements, which were taken for selected gate voltages between −2 and 2 V. Although the damping of the plasma waves is faster than that for Device 1, the extracted plasma speeds are of comparable values. They also show the expected minimum towards $V_{g,CNP}$.

In conclusion, we directly observed plasma waves in the channel of graphene TeraFET detectors (TeraFETs: antenna-coupled field-effect transistors for THz detection) when these were illuminated by narrow-band pulses of radiation at 2 THz from a free-electron laser. This visualization of plasma waves was made possible by the use of TeraFETs with a buried gate configuration, which leaves the graphene sheet accessible for near-field probing with an s-SNOM. The asymmetric antenna design enforced injection of the plasma waves from the source side into the transistor channel. The plasma-wave signal was found to sit on a pedestal arising from the partially screened THz electric field of the gate electrode. The plasma waves were found to be overdamped, with a propagation distance of several hundred nanometers and a short sub-100-fs lifetime, in good agreement with theoretical expectations. At gate voltages sufficiently far away from the Dirac point, where fluctuations of the potential of the exposed graphene sheet were significant, the propagation speed of the plasma waves exhibited the expected weak dependence on the gate voltage. Indications for wave scattering at surface contaminations could be identified. The formation of standing plasma waves was not observed because the channel length (1.8 μm) was larger than the propagation distance. Future work will focus on the observation of these standing waves and on measurements at higher THz frequencies that were not accessible with the given device structure with sufficiently good data quality because of limitations due to the antenna and the detector in combination with the elevated noise level of the free-electron laser radiation.

## Methods

Basic experimental aspects of the s-SNOM measurements are described here. For more information concerning (i) device fabrication, (ii) the choice of the radiation frequency, (iii) the fitting model for the s-SNOM line scans, (iv) the distinction of signal contributions and the s-SNOM data processing, (v) simulations of the possible influence of graphene surface contaminations on the s-SNOM signal, (vi) simulations of the propagating plasma waves (see ‘Movie-2THz.mp4’ and ‘Movie-4THz.mp4’) and of the s-SNOM signal to be expected, (vii) the additional figures mentioned in the text, and (viii) error calculations of the fits of the s-SNOM data, consult the Supplementary Materials.

### s-SNOM measurements

Our home-made s-SNOM^47^ is based on an atomic force microscope (AFM) with a PtIr-coated cantilever probe tip, which was operated in tapping mode with a commercial RHK R9 AFM controller. The tapping amplitude of the commercial probe tips (Nanosensors PPP-NCLPt) was ~100 nm, with an oscillation frequency of 190 kHz. The sample and the tip were illuminated by the radiation of the free-electron laser FELBE at Helmholtz-Zentrum Dresden-Rossendorf, Germany, which is a tuneable pulsed laser source operating over a wavelength range of 4–250 µm. It delivers pulses with a wavelength-dependent duration of 1–20 ps (10 ps at 2 THz) at a repetition rate of 13 MHz. In this work, it was operated at a radiation frequency of 2 THz with a time-averaged laser power of 45 mW (pulse energy of 3.46 nJ, peak power of 346 W). The beam was coupled into the setup, propagating perpendicular to the bow-tie antenna’s symmetry axis, with s-polarization of the radiation to achieve the best coupling to the antenna. The beam was split into two parts by a geometrical beam-splitter (gold-coated silicon wafer). One part was focused onto the probe tip with a paraboloidal mirror (focal length: 7.5 cm). The other part was directed onto a power meter to constantly monitor the incident power. The radiation backscattered from the probe tip was filtered with a p-polarized analyser before it was detected with a fast liquid-helium-cooled InSb hot-electron bolometer by QMC Instruments Ltd. The detector signal was fed into the R9 AFM controller and processed with higher-harmonic demodulation^47^. The THz beam path was purged with nitrogen to suppress absorption by water vapor in the air. Prior to the s-SNOM measurements, the setup was aligned, and its performance was tested with a plain gold sample. Fig. S9 shows typical s-SNOM data for this case, demodulated at the first, second, and third harmonic of the AFM cantilever oscillation frequency. The curves exhibit signal decay with increasing tip-sample distance. The signal-to-noise ratio was ~40, as measured with the sheet-gold reference sample, and was determined by the FELBE radiation source, which carries one to two orders of magnitude more noise than a table-top laser. The decay length of the signal decreased for higher harmonics, which scales with the improvement in the spatial resolution of the near-field scan. The decay of the length to half of the initial value at the sample surface was reached after 17 nm for the third harmonic demodulation. Details regarding the s-SNOM operation with FEL radiation in the THz range are presented in ref. ^47^.

## Supplementary information

Supplementary Information

Movie-2THz

Movie-4THz
