# Supplementary material for: Direct nanoscopic observation of plasma waves in the channel of a graphene field-effect transistor
Source: Light Sci Appl. 2020 Jun 4;9:97. doi: 10.1038/s41377-020-0321-0 (PMC7272618; doi:10.1038/s41377-020-0321-0)
Supplement: Supplementary file 1 — Supplementary Information [file 41377_2020_321_MOESM1_ESM.pdf]

Supplementary Information

Direct nanoscopic observation of plasma waves in the channel of a graphene field-effect transistor

Amin Soltani,[1, a,b)] Frederik Kuschewski,[2, a)] Marlene Bonmann,[3, a)] Andrey Generalov,[3, 4] Andrei Vorobiev,[3] Florian Ludwig,[1] Matthias M. Wiecha,[1] Dovilė Čibiraitė, [1] Frederik Walla,[1] Stephan Winnerl,[5] Susanne C. Kehr,[2] Lukas M. Eng,[2, 6] Jan Stake,[3] and Hartmut G. Roskos[1, b)]

[1)] *Physikalisches Institut, Johann Wolfgang Goethe-Universität, Max-von-Laue-Str. 1, D-60438 Frankfurt am Main, Germany*

[2)] *Institut für Angewandte Physik, Technische Universität Dresden, Nöthnitzer Str. 61, D-01187 Dresden, Germany*

[3)] *Department of Microtechnology and Nanoscience, Chalmers University of Technology, SE-41296 Gothenburg, Sweden*

[4)] *Present address: Department of Electronics and Nanoengineering, Aalto University, Tietotie 3, 02150, Espoo, Finland*

[5)] *Institute of Ion Beam Physics and Materials Research, Helmholtz-Zentrum Dresden-Rossendorf, Bautzner Landstraße 400, D-01328 Dresden, Germany*

[6)] *Complexity and Topology in Quantum Matter (CT.QMAT), Cluster of Excellence EXC 2147, Dresden/Würzburg, Germany*

[a)] These three authors contributed equally.

[b)] Electronic mail: roskos@physik.uni-frankfurt.de, soltani@physik.uni-frankfurt.de

➢ Device fabrication

Figure S1a shows the layout of the GFET photodetectors used in this work. The devices were fabricated on 280 µm-thick highly resistive silicon substrates after growth of a 300 nm-thick SiO_2 layer by thermal oxidation.

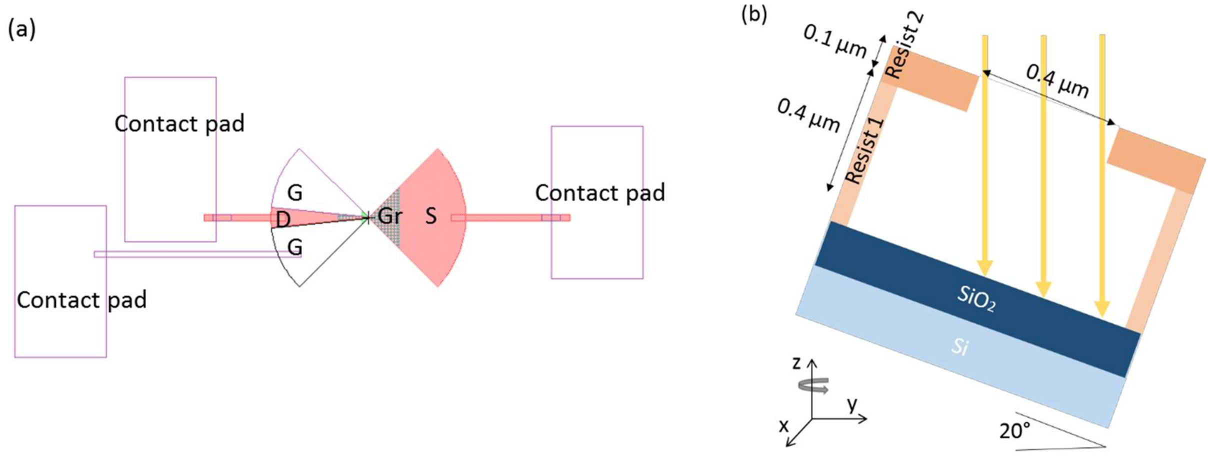

Figure S1: (a) Layout of a GFET detector with labelled gate (G), source (S), and drain (D) contacts, and a graphene (Gr) mesa. (b) Illustration of the evaporation step of the bottom gate. A slow evaporation rate together with a fast rotation of the sample holder containing the tilted substrate resulted in sloped sidewalls of the bottom gate.

The fabrication steps are illustrated in Fig. S2. First, the gate was patterned by e-beam lithography followed by the deposition of 2 nm of Ti and 20 nm of Au by e-beam evaporation and standard lift-off (Fig. S2a). The gate was created with sloped sidewalls to avoid abrupt gate boundaries. This allowed the graphene film to cover the gate without being suspended in the access areas between the gate edges and the source/drain contacts, thereby avoiding the risk of breaking the graphene film and ensuring a better transmission of the plasma waves from the source to the gated region. The sloped sidewalls were formed by evaporating the Au layer with a deposition rate of approximately 0.8 $Ås^{-1}$ while tilting the substrate at 20° and rotating it with 4 revolutions per second as illustrated in Fig. S1b. In the scanning-electron micrograph (SEM) image of the gate metallization in Fig. S3, the sloped sidewalls of the gate are seen as the darker areas at the periphery. In the next step, a 25 nm-thick Al_2O_3 layer was grown at 300°C by atomic layer deposition (Fig. S2b). Then, easy-transfer graphene, supplied by Graphenea, was transferred from a sacrificial polymer layer onto the substrate (Fig. S2c). The GFET channel was patterned by e-beam lithography followed by oxygen plasma etching (Fig. S2d)). In the next step, the parts of the bow-tie antenna which constitute the drain/source contacts and the contact pads were patterned by e-beam lithography followed by e-beam evaporation of 2 nm Ti / 10 nm Pd / 150 nm Au and standard lift-off (Fig. SI.2e). Next, the gate contact part of the bow-tie antenna was patterned by e-beam lithography. Before the gate metal was deposited, the Al_2O_3 was etched away in the gate contact area using photolithography and chemical treatment by buffered oxide etchant (BOE:water 1:10) to provide electrical contact between the gate and the top gate contact metal. The latter was then deposited by e-beam evaporation of 4 nm Ti and 270 nm Au followed by standard lift-off (Fig. S2f)).

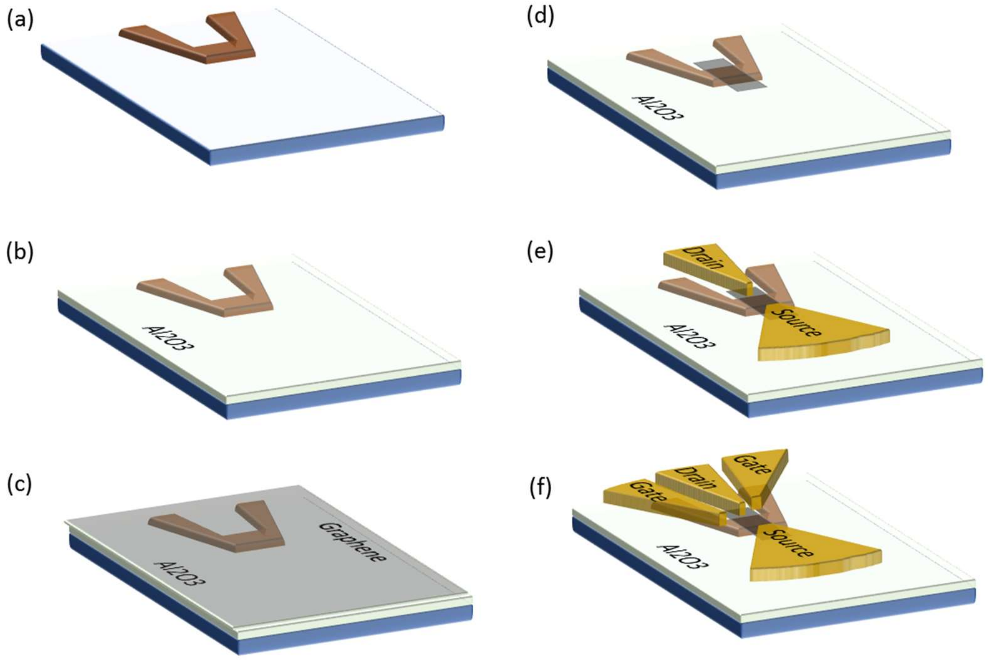

Figure S2: Fabrication steps of the GFET detectors. (a) Formation of the gate with sloped sidewalls. (b) Growth of Al_2O_3 by thermal atomic layer deposition at 300°C. (c) Transfer of the graphene film onto the substrate. (d) Patterning of the graphene channel by e-beam lithography and oxygen plasma etching. (e) Deposition and patterning of the drain and source contacts, and contact pads (contact pads are not shown). (f) Deposition and patterning of the gate contact, and contact pads (contact pads are not shown).

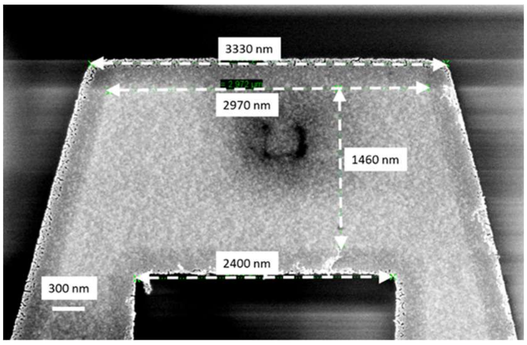

Figure S3: SEM image of the GFET's gate with the sloped sidewalls.

➢ Choice of radiation frequency

Plasma waves, according to theory (see [6] and references of the main text), are expected in the FET's channel if kL > 1, with k being the wave-vector of the plasmon polariton and L the gate length (here, L = 1.8 μm. With the linear dispersion relation ω = sk, s being the (gate-voltage-dependent) plasma wave velocity, one estimates for the usual case of $s > v_F = 10^6$ ms^{-1} (v_F: Fermi velocity) for our carrier densities, that plasma waves are to be expected for frequencies f = ω/(2π) > s/(2πL) = 0.5/(2π) THz = 80 GHz. Plasma waves are hence expected already at rather low frequencies in our long-channel graphene transistors. The waves are, however, overdamped if ωτ < 1, which, for τ ≅ 70 fs, is the case for f < 2.3 THz (see also Sec. "Simulation of the plasma wave and the expected s-SNOM signal simulations" below). This implies that one would like to measure at about 2 THz and higher.

We performed measurements at 1.3 THz, 2.0 THz and at 3-4 THz. The data for 1.3 THz do not provide more insight than those measured at 2.0 THz, which led us to decide to only present those for 2.0 THz. On the other hand, the data quality of the measurements at 3 THz and above turned out to be too poor. We attribute this to the combined effect of a performance decrease with rising frequency of both the detector and the antenna. The latter, with its split drain/gate leaf, exhibits optimal performance at around 2 THz. Antenna simulations indicate that, with frequency rising above 2 THz, the capacitive coupling of the drain and gate leafs increasingly becomes inductive, which leads to a rapid decrease of the rectified signal above 2 THz. As a result of both, the signal-to-noise ratio of the s-SNOM measurements was intolerably low at the measurements which we performed at 3 THz and above. For this reason, we decided to present only the 2.0 THz data in this paper.

➢ Fitting procedure

In the following text, we explain the modeling of the results of the near-field s-SNOM experiments. In the self-homodyne detection scheme employed here[1,2], the detected signal (prior to demodulation on the basis of the cantilever' oscillation frequency) is proportional to the intensity of the total, complex-valued electric field scattered to the detector. Not all of this signal comes from the probe tip. One identifies two main contributions,

$$E_{total} = E_b e^{i\phi_b} + E_{near-field} \quad \text{.} \qquad \textit{Eq. 1}$$

$E_{near-fiel}$ denotes the scattered field originating from local or guided THz fields, and E_b is the background electric field caused by reflections and scattering at objects in the pathway of the incident light (including the sample and the cantilever) and manifesting itself often by the formation of spurious standing waves. ϕ_b denotes the phase difference of the background signal relative to the incident field. $E_{near-field}$ includes two terms,

$$E_{near-field} = E_m + E_{pl} \quad \text{.} \qquad \textit{Eq. 2}$$

Here, E_m represents the material-related near-field component, which probes the local dielectric response of the sample underneath the tip as often described by the image dipole model[3]. This component particularly contains (i) contributions by the graphene sheet and (ii) contributions by the gate electrode, which are not entirely screened by the graphene layer. The latter includes the (partially screened) amplitude of the THz signal on the gate electrode which is the main contributing factor to the pedestal

discussed upon presentation of Fig. 2(b) in the main text. Note, that the DC gate bias - unlike the THz signal on the gate - does not contribute directly (i.e., other than by modification of the material properties) to the near-field signal because it does not produce scattered radiation at THz frequencies which the detector can record. E_{pl} is the electric field of the plasma wave launched from the source contact and propagating (and decaying) along the channel, here the x direction. Fig. S4 illustrates the various electric fields.

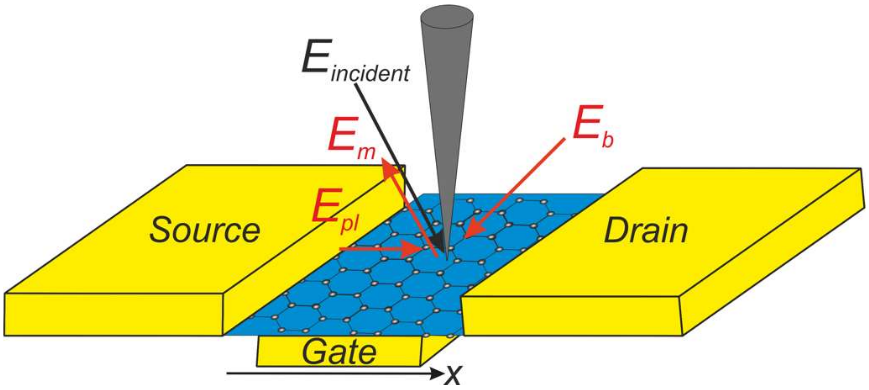

Figure S4: Different components of the total electric field on the gated channel probed by the s-SNOM's metal tip (plotted in grey).

In the following, we assume that all contributions except for E_{pl} are spatially invariant across the gated channel along the direction of the line scans described in the main body of the paper. The weak spatial modulations, which we observe in addition to the plasmon-related features in the experiments, are tentatively attributed to scattering of the plasma waves at surface defects, see below. These variations are not taken into account in the fitting model described here.

E_{total} now reads as

$$E_{total} = E_b e^{i\phi_b} + E_m + E_1 e^{-i(k'_{pl}x+\phi_{pl})} e^{-k''_{pl}x} \qquad \text{Eq. 3}$$

Here, $E_1 e^{-i(k'_{pl}x+\phi_{pl})} e^{-k''_{pl}x}$ represents the plasma wave, with ϕ_{pl} being its phase retardation relative to the incoming radiation. The plasma waves exhibit a linear dispersion relation[4], with the complex-valued wave vector (k'_{pl}, k''_{pl}) given by[5]

$$k'_{pl} = \frac{\omega}{v_{pl}}\left(\frac{(1+\omega^{-2}\tau^{-2})^{\frac{1}{2}}+1}{2}\right)^{\frac{1}{2}}, \quad k''_{pl} = \frac{\omega}{v_{pl}}\left(\frac{(1+\omega^{-2}\tau^{-2})^{\frac{1}{2}}-1}{2}\right)^{\frac{1}{2}}, \qquad \text{Eq. 4}$$

where v_{pl} and τ are the speed of the plasma wave and the scattering time (momentum relaxation time), respectively. The total intensity, proportional to the detected signal in the s-SNOM experiments, reads as

$$I_{total} = E_{total} \times E^*_{total} = \left(E_b e^{i\phi_b} + E_m + E_1 e^{-i(k'_{pl}x+\phi_{pl})} e^{-k''_{pl}x}\right) \times \left(E_b e^{-i\phi_b} + E_m + E_1 e^{i(k'_{pl}x+\phi_{pl})} e^{-k''_{pl}x}\right) = E_b^2 + E_b E_m e^{i\phi_b} + E_b E_1 e^{i\phi_b} e^{i(k'_{pl}x+\phi_{pl})} e^{-k''_{pl}x} + E_m E_b e^{-i\phi_b} + E_m^2 + E_m E_1 e^{i\left(k'_{pl}x+\phi_{pl}\right)} e^{-k''_{pl}x} + E_1 E_b e^{-i\phi_b} e^{-i(k'_{pl}x+\phi_{pl})} e^{-k''_{pl}x} + E_m E_1 e^{-i(k'_{pl}x+\phi_{pl})} e^{-k''_{pl}x} + E_1^2 e^{-2k''_{pl}x}. \qquad \text{Eq. 5}$$

In order to reject contributions other than the pure near-field signals as much as possible, one only keeps signal contributions, which are modulated at a higher harmonic of the AFM cantilever's oscillation frequency[4,5]. This eliminates the E_b^2 term. As the background field E_b is much larger than the involved near-fields (E_m, E_{pl}), only mixed terms with E_b remain. As a result, the detected intensity after higher harmonic demodulation reads

$$I_{total} \sim 2E_m E_b \cos(\phi_b) + 2E_1 e^{-k''_{pl}x} E_b \cos\left(k'_{pl}x + \phi_{pl} + \phi_b\right) . \quad \textit{Eq. 6}$$

In the fitting procedure, we set the phase ϕ_b to zero. Then

$$I_{total} \sim 2E_b\left(E_m + E_1 \cos\left(k'_{pl}x + \phi_{pl}\right) e^{-k''_{pl}x}\right) . \quad \textit{Eq. 7}$$

The fitting curves shown in Fig. 2(c) and the results of the fitting procedures displayed in Figs. 2(d) and 3(b) of the main paper were obtained with Eq. 7 by employing a nonlinear least-square solver. The five independent fit parameters are E_m, E_1, k'_{pl}, k''_{pl} and ϕ_{pl}. E_b is not independent as it appears only in product terms with E_m and E_1, and hence was fixed to $2E_b = 1$. k'_{pl} and k''_{pl} are dependent on v_{pl} and τ (Eq. 4) and therefore were replaced by v_{pl} and τ as independent fit parameters.

The specifics of the measured curves (see Fig. 2(c)) allowed us to define reasonable starting values for several fit parameters: (i) Assuming complete decay of the plasma wave at the drain-sided end of the channel ($x = L$, with L being the length of the channel) leads to

$$I_{total}(x = L) \sim E_m . \quad \textit{Eq. 8}$$

This means that the pedestal height of the respective measured data allowed us to define an initial guess for E_m. (ii) At the source-sided end of the channel ($x = 0$) where the plasma wave was launched, Eq. 7 now reads

$$I_{total(x=0)} \sim E_m + E_1 \cos\left(\phi_{pl}\right). \quad \textit{Eq. 9}$$

With the initial value of E_m fixed, the estimated amplitude of the plasma wave now provided an initial guess for $E_1 \cos\left(\phi_{pl}\right)$. For ϕ_{pl}, we chose the starting value $\phi_{pl} = 0$. It is worth mentioning that we varied the initial guess of ϕ_{pl} several time, which did not lead to different results.

Finally, the initial guess for the plasma wave velocity v_{pl} was $3 \times v_F$. For the scattering time τ, we selected a value from within the range of accepted values for CVD graphene and kept the value fixed during the fitting process. Varying the scattering time and repeating the fitting procedure led to the gate-voltage-dependent results presented in Fig. 2(d) and Fig. 3(b), with the plotted propagation speed of the plasma wave being a result of the curve fitting for the respective scattering time. For a discussion of the fitting errors, find more information below in the final chapter of this Supplementary.

➢ Distinction between the signal contributions of the plasma wave and of the pedestal produced by the THz signal on the gate electrode

The main constituent of the near-field signal is the sum of the THz signal on the gate electrode and the smaller contribution from the plasma wave on the graphene sheet. In the fitting procedure described above, the former is contained in the term E_m and the latter is represented by E_{pl} with the amplitude E_1.

Here, we summarize how the two contributions can be distinguished in the measured near-field traces. We have four indicators which allow us to identify the plasma wave and to separate it from the THz pedestal:

1. The antenna was designed such that the plasma wave is launched only from the source side of the transistor. No injection is expected from the drain side.
2. Theory predicts that the plasma wave is damped with the typical sub-100 fs scattering time of the electrons in graphene which is deposited on an oxide material and exposed to air.
3. The THz signal on the gate electrode contributes with a spatially constant signal (pedestal) in the s-SNOM measurements because the graphene monolayer screens this voltage only partly, an effect well known in the literature.
4. The plasma wave's velocity is gate-voltage-dependent, while the pedestal is not expected to exhibit a voltage dependence.

With these four features, we can unambiguously distinguish the two contributions.

➢ s-SNOM data recording and post-processing

To reduce noise in the line scans, the lock-in integration time was set to 1 s (while the time for each scan was chosen to be 100 s). The results of three scans were averaged to further minimize statistical fluctuations in the data. Forward and backward scan directions were handled separately. The line traces shown in Fig. 2 in the paper represent only averaged forward scans. We used the backward traces however for a comparison with the forward traces. We observed small shifts of the positions of the features for the two scan directions which resulted from the creep of the open-loop piezoelectric scanners. To eliminate these drifts of the s-SNOM curves, the simultaneously recorded AFM data was used as a reference to shift the curves for a proper overlay. Comparing now the features of the near-field signals in detail, we found them to be near-identical. Artefacts, which may in principle arise from slow rise and fall times of the signal of the lock-in amplifier, were not observed; the chosen integration time had no detrimental effect.

➢ Scattering of the plasma waves at graphene surface contaminations

The line scans over the gated graphene layer exhibit weak modulations not associated with the plasma waves, as can be seen in Figs. 2(b) and (c) in the main body of this work. The signatures are stable during a measurement but vary for different locations of the line scans along the TeraFET's channel. Here, we show by simulations that similar modulations of the s-SNOM signal are obtained if we include the scattering of the plasma waves at surface contaminations or imperfections of the graphene sheet into the picture. That such defects are found on the devices is seen in Fig. S5 displaying a SEM image of a device's channel region. The image was taken after the s-SNOM measurements. Several point-defect-like imper-fections are visible on the gate, with dimensions below 100 nm and distributed randomly. The nature of the unwanted objects is unclear, but we speculate that many of them are gold droplets deposited during device fabrication. The reason for this assumption is that similar droplet-like contaminations were observed in subsequent fabrication runs of new devices where freshly prepared gate electrodes were inspected by AFM measurements directly after processing step a) of Fig. S2. However, we cannot exclude that some of the contaminants seen in Fig. S5 are dust particles attracted to the graphene layer by the applied electrostatic gate voltage during the near-field measurements, which take place in open air.

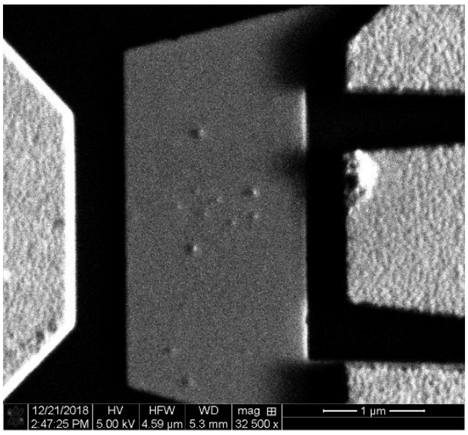

Figure S5: SEM image of the device, which shows several point-like imperfections in the channel. These can act as scattering centers for the plasma wave impinging from the side of the source contact. The SEM image was collected after the near-field measurements.

While we avoided taking s-SNOM line scans directly across the objects (whose presence reveals itself in the AFM data recorded together with the s-SNOM signal during the line scans), they could still influence the data via wave scattering effects causing interference signatures. To explore this idea, we performed full-wave simulations for the case of surface plasmon polaritons (SPPs) propagating on a gold surface. This calculation was performed with the CST Maxwell solver software (CST: Computer Simulation Technology, of Dassault Systèmes). Fig. S6a shows the modeled structure with four randomly distributed scatterers on the gold surface. They consist of gold spheres with different radii as specified in the figure caption.

(a)

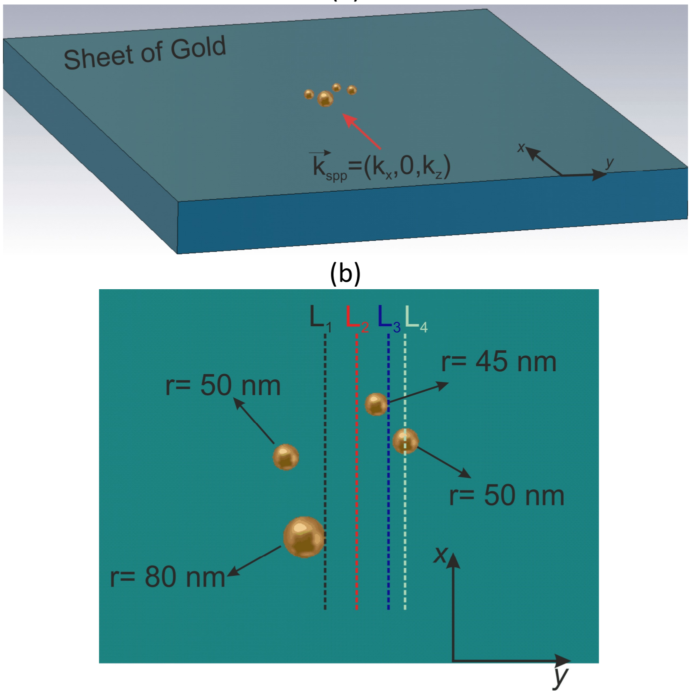

Figure S6: (a) The structure employed for the CST simulation. Four gold spheres (with the radii being 80 nm, 50 nm, 50 nm and 45 nm) are randomly located on a sheet of gold. Note the coordinate system displayed on the right side of the plot. (b) Locations of the line scans shown in Fig. SI.7. SPP are generated at the bottom edge of the gold sheet and propagate in +y-direction. The electric-field plots in Fig. SI.7 are along the four vertical lines drawn in different colors.

Fig. S7 shows the absolute value of E_z along the different lines of Fig. S6b for the case of (a) no scatterers on the gold sheet (reference) and (b) for the case of the four scatters being located on the gold. The reference shows identical decay behavior of the four curves, denoted as L1 to L4, with no modulations on them, while the curves in (b) exhibit modulations on top of the SPP decay resulting from interference of scattered and unscattered fields. The modulations vary from L1 to L4. We note that L2 is in a region without any scatterer (a situation like in our line scans on the graphene TeraFETs), but still shows an interference pattern.

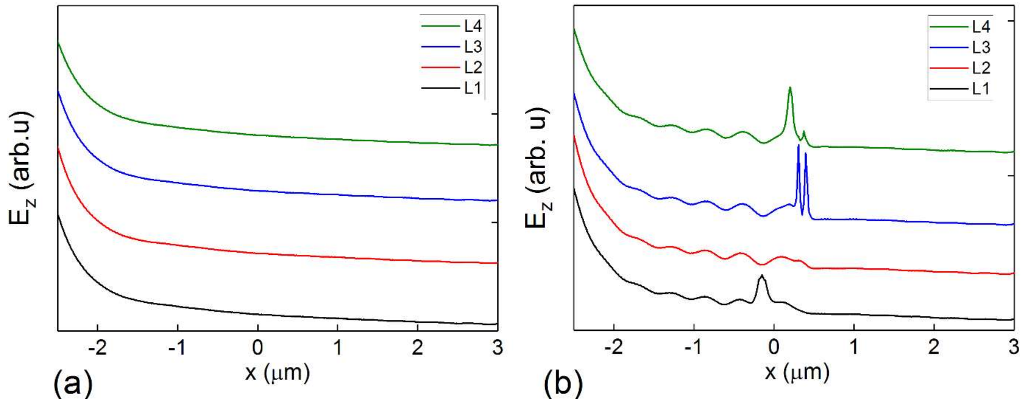

Figure S7: (a) z-component of the electric field E_z along the lines presented in Fig. S6 for the reference case, (b) E_z for the presence of four scatters. Only the blue curves are scaled according to the simulation results, the other curves are shifted vertically for clarity.

➢ Simulation of the plasma wave and the expected s-SNOM signal

We simulated the propagation of a plasma wave on the FET's gated graphene on the basis of Eq. 11 and 12 of Ref. 6 using the geometrical parameters of the FET's channel as given in the main paper. It is assumed that the wave is launched at x = 0, simulating the source-sided in-coupling of the THz signal of the experiment. The movies provided as supplementary information display the evolution of the plasma wave assuming a scattering time of τ of 50 fs and a gate voltage swing of (V_g - $V_{g,CNP}$) of 0.35 V, which yields a plasma wave velocity v_{Pl} = 3.5×v_F. "Movie-2THz.mp4" assumes excitation at 2.0 THz, "Movie-4THz.mp4" one at 4.0 THz, the former one hence displaying an overdamped case (with $\omega\tau$ = 0.63), the latter an oscillatory solution (with $\omega\tau$ = 1.26).

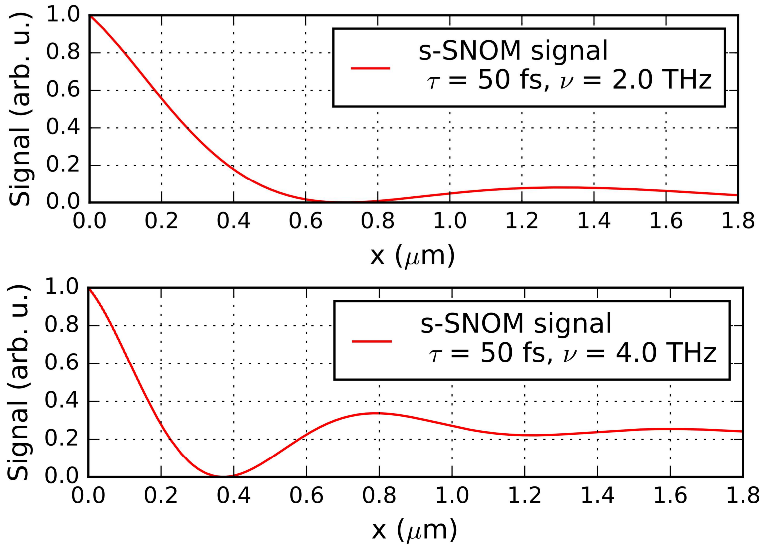

Figure S8: (a) Simulated s-SNOM signal for excitation of a plasma wave at 2.0 THz (upper panel) and 4.0 THz (lower panel). The wave is assumed to be launched at x = 0 μm.

Fig. S8 shows the expected s-SNOM signal for these two cases in the steady-state situation. It was calculated by the determination of the time-averaged power of the interference signal of the electric fields of the plasma wave and of a radiation field impinging perpendicularly onto the graphene (simulating the excitation scheme of the experiment with the phase front of the illuminating THz beam being parallel to the TeraFET's antenna axis). The graphs indicate that the wave character of the plasma wave is easier to discern at the higher frequency. Unfortunately, respective measurements with the FEL beam did not result in a signal quality sufficiently good for further analysis.

➢ Supplementary graphs

Figure S9 displays the approach curves (detected s-SNOM signal as a function of distance of the cantilever's tip from the sample's surface) measured on a 100 nm thick gold film deposited on a silicon substrate for demodulation at the 1st, 2nd and 3rd harmonic of the cantilever's oscillation frequency. The grey area marks the region over which the signal decays to half the value for the case of full contact. This range, named as active area, is often used to define the lateral spatial resolution of the measurements[2] as it scaled with the effective tip apex curvature. It decreases from 50 nm for the 1st harmonic to 25 nm for the 2nd and to 20 nm for the 3rd harmonic.

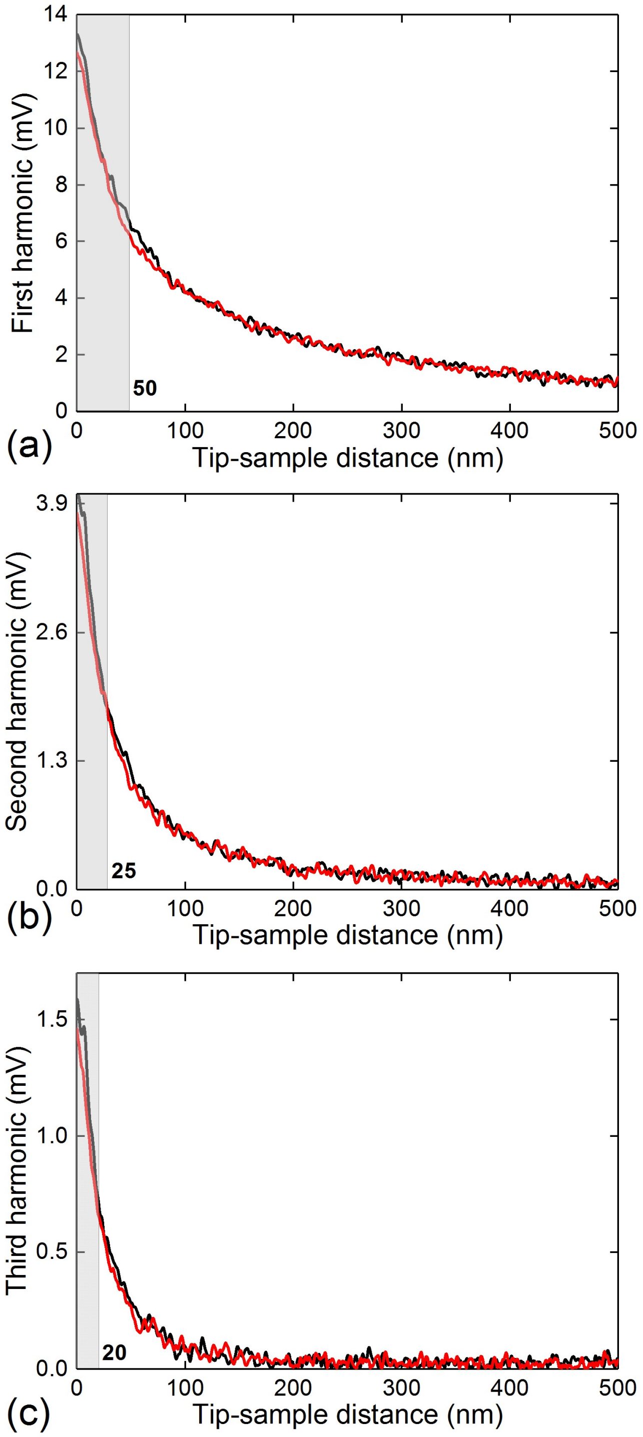

Figure S9: Approach curves of the cantilever in the s-SNOM experiments under illumination by 2-THz radiation. The plots in (a)-(c) show the measured signal strength as a function of the tip-sample distance for the cases of demodulation at the first, second and third AFM cantilever oscillation frequency, respectively. Red and black curves show the downward and upward approach curve measurement respectively. The active distance reduced from 50 nm to 20 nm, which confirms that the demodulation at higher harmonics suppresses more of the background influence.

With the data of Fig. S9, we can estimate the signal-to-noise (SNR) ratio of the measurement. By taking the signal value in close approximation of the tip to the sample and divide it by background noise level (at large distances), we obtain a value of the SNR of about 40 (30 to 50 depending on the order of the higher-harmonic demodulation). The noise level is mostly determined by the noise level of the laser source.

Figure S10 is a supplement of Figs. 1(b) and (d) of the main text. While Fig. 1(b) displays an SEM image of the surroundings of the TeraFET's gated channel region, and Fig. 1(d) shows an s-SNOM image of the second harmonic, Fig. S10 exhibits the corresponding AFM image (Fig. S10a) recorded simultaneously with the s-SNOM images at the various harmonics (Fig. S10b,c,d). The buried gate electrode is slightly visible in the false-color scale of the AFM image.

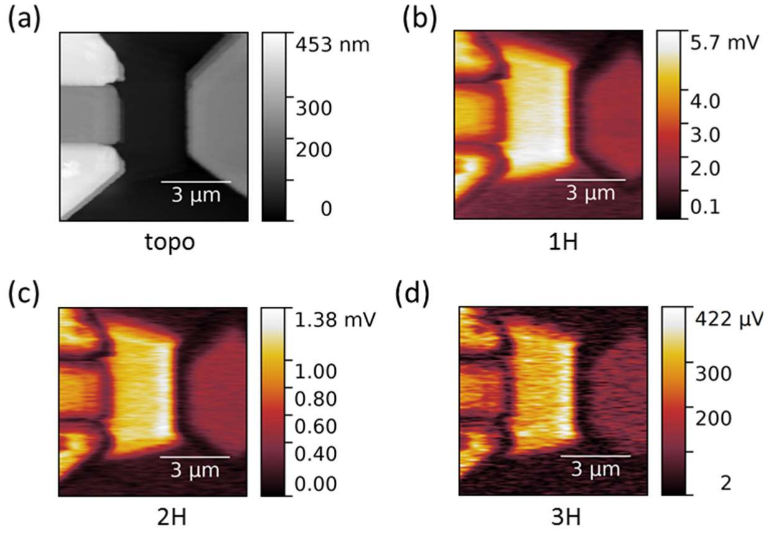

Figure S10: AFM image of the gated-channel region, for which the s-SNOM signal is presented in Fig. 1(d) of the main text. The source contact is on the right side, the drain and gate contacts on the left (cf. Fig. 1).

➢ Error calculations of the fit procedure of the s-SNOM traces

When fitting the s-SNOM traces (of Device 1 shown in Fig. 2(c) and the corresponding data of Device 2 leading to the plot shown in Fig. 3(b)) with Eq. 7, we assumed – as described in the main text – a value of τ from the range 40-70 fs for Device 1, resp. 25-60 fs for Device 2, and obtained a value for the speed v_{pl} of the plasma wave for each chosen value of τ. For the fitting procedure, we used the nonlinear curve-fitting (data-fitting) routine of MATLAB which is based on the least-squares method (for details, see https://de.mathworks.com/help/optim/ug/lsqcurvefit.html). For this technique, MATLAB offers the possibility to calculate the confidence intervals of the nonlinear regression parameter, as described in https://de.mathworks.com/help/stats/nlparci.html?searchHighlight=nlparci&s_tid=doc_srchtitle. This routine was used for the determination of the confidence intervals of our data fits.

Fig. S11 shows in an exemplary way the fit curve (in blue color) to the experimental s-SNOM trace (brown curve) of Device 1 measured at a gate voltage of -3 V for a value of τ of 55 fs. The same data are displayed at the bottom of Fig. 2(c) in the main text. In Fig. S11, however, we added the 90%-confidence intervals as

horizontal blue bars to the theoretical curve. The confidence range is remarkably narrow, which indicates that the outcome of the fitting process is reliable and reproducible within the range of options of the fit function of Eq. 7.

Fig. S12 displays the error bars (90%-confidence ranges) of v_{pl} for all gate voltages of our measurements on Device 1 for three values of τ. The error bars are so short that they can hardly be distinguished from the v_{pl} data points. While these findings underscore the reliability of the curve fits, they also indicate that one obtains good fits (i.e., suitable values for the pair of parameters τ and v_{pl}) over a large range of assumed values of τ. An independent measurement of τ is required to determined v_{pl} unambiguously.

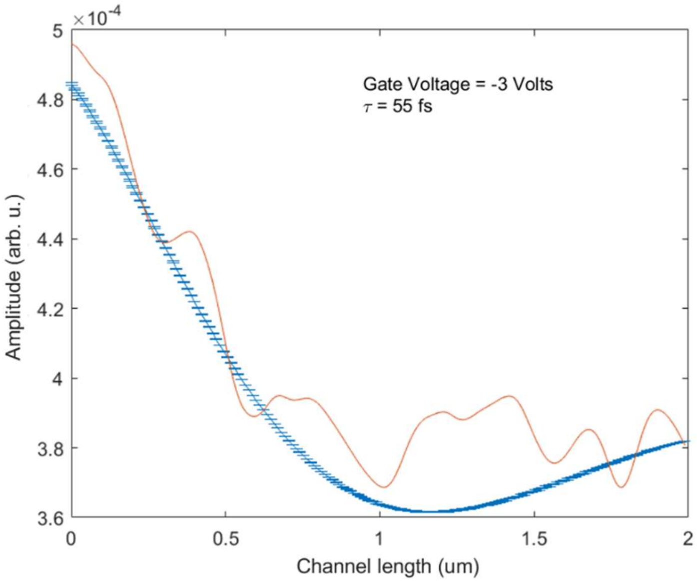

Figure S11: The s-SNOM trace of Fig. 1(c) (bottom) – experiment (brown curve) and theory (blue curve) – with the 90%-confidence range given by the horizontal blue bars.

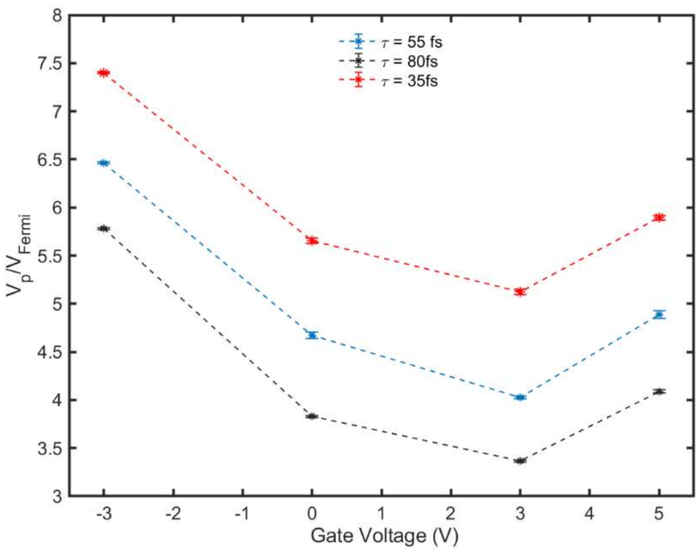

Figure S12: Results of the fitting of the s-SNOM traces of Device 1: Resultant values of the plasma velocity as a function of the gate voltage for three values of the damping time. Vertical error bars are plotted indicating the 90%-confidence intervals of the fits.

References

1. Knoll, B. & Keilmann, F. Enhanced dielectric contrast in scattering-type scanning near-field optical microscopy. *Opt. Commun.* **182,** 321–328 (2000).
2. Kuschewski, F. *et al.* Narrow-band near-field nanoscopy in the spectral range from 1.3 to 8.5 THz. *Appl. Phys. Lett.* **108,** 113102 (2016).
3. Cvitkovic, A., Ocelic, N. & Hillenbrand, R. Analytical model for quantitative prediction of material contrasts in scattering-type near-field optical microscopy. *Opt. Express* **15,** 8550 (2007).
4. Ocelic, N., Huber, A. & Hillenbrand, R. Pseudoheterodyne detection for background-free near-field spectroscopy. *Appl. Phys. Lett.* **89,** 101124 (2006).
5. Walla, F. *et al.* Anisotropic excitation of surface plasmon polaritons on a metal film by a scattering-type scanning near-field microscope with a non-rotationally-symmetric probe tip. *Nanophotonics* **7,** 269–276 (2018).
6. Tomadin, A.& Polini, M. Theory of the plasma-wave photoresponse of a gated graphene sheet. *Phys. Rev. B* **88**, 205426 (2013).
